# Concordance of Computed Tomography Regional Body Composition Analysis Using a Fully Automated Open-Source Neural Network versus a Reference Semi-Automated Program with Manual Correction

**DOI:** 10.3390/s22093357

**Published:** 2022-04-27

**Authors:** Sandra L. Gomez-Perez, Yanyu Zhang, Cecily Byrne, Connor Wakefield, Thomas Geesey, Joy Sclamberg, Sarah Peterson

**Affiliations:** 1 Department of Clinical Nutrition, Rush University, Chicago, IL 60612, USA; sarah_j_peterson@rush.edu; 2 Rush Bioinformatics and Biostatistics Core, Rush University Medical Center, Chicago, IL 60612, USA; yanyu_zhang@rush.edu; 3 Department of Kinesiology and Nutrition, University of Illinois at Chicago, Chicago, IL 60612, USA; cbyrne7@uic.edu; 4 Department of Internal Medicine, Brooke Army Medical Center, Fort Sam Houston, TX 78234, USA; connor_j_wakefield.mil@mail.mil; 5 Department of Diagnostic Radiology and Nuclear Medicine, Rush University Medical Center, Chicago, IL 60612, USA; thomas_c_geesey@rush.edu (T.G.); joy_s_sclamberg@rush.edu (J.S.)

**Keywords:** computed tomography, body composition, validation, agreement, adipose tissue, muscle, automated segmentation, artificial intelligence

## Abstract

Quick, efficient, fully automated open-source programs to segment muscle and adipose tissues from computed tomography (CT) images would be a great contribution to body composition research. This study examined the concordance of cross-sectional areas (CSA) and densities for muscle, visceral adipose tissue (VAT), subcutaneous adipose tissue (SAT), and intramuscular adipose tissue (IMAT) from CT images at the third lumbar (L3) between an automated neural network (test method) and a semi-automatic human-based program (reference method). Concordance was further evaluated by disease status, sex, race/ethnicity, BMI categories. Agreement statistics applied included Lin’s Concordance (CCC), Spearman correlation coefficient (SCC), Sorensen dice-similarity coefficient (DSC), and Bland–Altman plots with limits of agreement (LOA) within 1.96 standard deviation. A total of 420 images from a diverse cohort of patients (60.35 ± 10.92 years; body mass index (BMI) of 28.77 ± 7.04 kg/m^2^; 55% female; 53% Black) were included in this study. About 30% of patients were healthy (i.e., received a CT scan for acute illness or pre-surgical donor work-up), while another 30% had a diagnosis of colorectal cancer. The CCC, SCC, and DSC estimates for muscle, VAT, SAT were all greater than 0.80 (>0.80 indicates good performance). Agreement analysis by diagnosis showed good performance for the test method except for critical illness (DSC 0.65–0.87). Bland–Altman plots revealed narrow LOA suggestive of good agreement despite minimal proportional bias around the zero-bias line for muscle, SAT, and IMAT CSA. The test method shows good performance and almost perfect concordance for L3 muscle, VAT, SAT, and IMAT per DSC estimates, and Bland–Altman plots even after stratification by sex, race/ethnicity, and BMI categories. Care must be taken to assess the density of the CT images from critically ill patients before applying the automated neural network (test method).

## 1. Introduction

Computed tomography (CT) images have been used for assessment of regional body composition in many clinical populations including cancer patients [1,2,3,4,5,6,7]. However, the technique for analyzing CT images is laborious, time-consuming, costly, and requires intensive training [8,9]. A commonly applied reference technique is a semi-automated method with human-analyzed correction of segmentation on CT imaging using the medical imaging software, SliceOmatic plus ABACS (TomoVision and Voronoi Health Analytics, Montreal, QC, Canada). The manual correction process takes approximately 15–20 min, which translates to approximately 1500 min or 60 h of work to analyze 100 CT images. Thus, the biggest limitation to CT body composition research is the lack of relatively easy and inexpensive programs or tools to detect and quantify muscle and adipose tissues at single or multiple spinal landmarks quickly and accurately. Various automated programs or neural networks have already been developed and tested (Paris, 2020, Automated body composition analysis of clinically acquired computed tomography scans using neural networks). A comprehensive list of neural networks with DSC estimates for various landmarks was recently published; however, most of these are not open-source programs. Paris et al. recently developed and validated a completely automated, neural-network, open-source framework called AutoMATiCA for the expeditious analysis of large repositories of CT images for cross-sectional areas (CSA) of various abdominal tissues (https://gitlab.com/Michael_Paris/AutoMATiCA accessed on 22 June 2021) [9]. In comparison to the human-analyzed technique, the AutoMATiCA program analyzes an image for various abdominal tissues in approximately 350 milliseconds per CT image, which translates to 35 min for complete analysis of 100 images.

In their paper, Paris et al. confirmed a high degree of agreement between this new program, AutoMATiCA (automated neural network), and a reference human-analyzed technique for CT images using the SliceOmatic software [9]. Following validation, Bland–Altman plots comparing neural-network analysis to human-analyzed CSA for different body composition parameters including muscle and various adipose tissues (intermuscular, visceral, subcutaneous) showed no biases (within limits of agreement). In addition, the authors reported strong agreement between the two methods using the Sorenson dice-similarity coefficient (DSC) of >0.80 [9,10]. For muscle, the DSC was 0.983 ± 0.013l between the human-analyzed technique and the network-predicted segmentation, for intermuscular adipose tissue (IMAT) it was 0.900 ± 0.034, for visceral adipose tissue (VAT), the DSC was 0.979 ± 0.019, and for subcutaneous adipose tissue (SAT), it was 0.986 ± 0.016 [9].

Given the need for validation among diverse patient populations and confirmation of the findings as reported by Paris et al., we tested the fully automatic program (automated neural network—AutoMATiCA) against SliceOmatic plus ABACS (reference method) with the human-based manual correction for analyzing CT images [9]. Additionally, the level of agreement using DSC and Bland–Altman plots as identified by Paris et al. needs to be further examined in subgroups of diverse communities: by sex, race/ethnicity, body mass index (BMI), and disease status. Thus, the purpose of this study was twofold: first, to confirm the concordance of a newly developed automated neural network (AutoMATiCA) for analyzing CT images for body composition at third lumbar (L3) vertebra with a reference semi-automatic human–based manual technique using the SliceOmatic plus ABACS Software; and second, to further examine the concordance between the two methods by disease status, sex, race/ethnicity, and BMI categories.

## 2. Materials and Methods

### 2.1. Study Design and Sample Population

A retrospective review was conducted on various adults at a major tertiary medical center who underwent CT abdominal imaging. Clinically acquired CT images obtained previously for research studies using electronic medical records of various adult medical and surgical patient populations (>18 years of age) were included. These images were previously analyzed with the reference software (SliceOmatic plus ABACS) using the semi-automatic plus human-based manual analysis technique as part of past research collaborations. CT images were obtained from retrospective cohort studies in various diverse clinical populations (breast cancer, 3%; COVID-19; 5%; colorectal cancer, 30%; critical illness, 9%; healthy controls, 31%; and metastatic breast cancer, 22%) at Rush University Medical Center (RUMC), University of Illinois at Chicago, and Loyola University Medical Center. CT images were previously anonymized. CT images included in this study were analyzed centrally by trained experts at RUMC Department of Clinical Nutrition. CT images were excluded if the images were poor quality or unevaluable (i.e., anasarca, poor positioning, excessive cutoffs, grainy, etc.). A total of 418 unique patients contributed 420 images for this study. Two patients provided two images to the study. The research studies providing anonymized CT images were previously approved according to institutional research review boards. Additionally, this study was reviewed and approved by the RUMC Institutional Review Board in accordance with protocols for human subject research.

The demographic and clinical variables collected for each adult patient were age (years), sex (male, female), race/ethnicity (White, Black, other, refused or unknown), clinical diagnosis, height (cm), weight (kg), and body mass index (BMI, kg/m^2^).

### 2.2. SliceOmatic Plus ABACS Analysis (Reference Method)

A total of 420 CT images at the third lumbar (L3) landmark previously analyzed for CSA and density of adipose tissues and muscle from clinically indicated CT scans were included in this study. Using standard protocols for abdominal body composition analysis, trained experts previously analyzed each single image to determine CSA (cm^2^) for SAT, VAT, IMAT, and muscle using medical imaging applications SliceOmatic plus ABACS v4.3 (TomoVision and Voronoi Health Analytics, Montreal, QC, Canada) [2,11]. The medical imaging software package permits segmental demarcation of each tissue compartment according to specific Hounsfield unit (HU). The HU scale from −1000 (representing air) to +2000 (representing bone) was developed by Sir Godfrey Hounsfield, inventor of the CT scanner, to measure the radiographic attenuation of images taken during a CT scan. The HU tissue specific threshold for VAT is −150 to −50 HU, for SAT and IMAT is −190 to −30 HU, and for muscle is −29 to 150 HU [2]. The tissue specific thresholds are preprogrammed. Density (mean HU) was also determined by the software for each adipose and skeletal tissue. Tissue boundaries were corrected manually as needed. The complete analysis of a single CT image took approximately 15–20 min. Intra-class coefficient of variations for body composition analysis for trained experts were previously examined and recorded to be less than 2%.

### 2.3. AutoMATiCA Analysis (Test Method)

AutoMATiCA conducts an automatic segmental analysis that yields estimates of cross-sectional area (CSA) and density (mean Hounsfield (HU) unit) of each adipose and muscle for each CT image at the L3 landmark [2]. To this end, the same 420 CT images analyzed segmentally with the reference method were processed for CSA (cm^2^) and tissue density using the test method, AutoMATiCA. Briefly, CT images were loaded into the program, a location to save the images and results was identified, and automated segmentation was performed the default segmentation determines CSA and density estimates for all the tissues at the L3 landmark (i.e., muscle, VAT, SAT, IMAT). Default HU ranges (HU thresholds of −150 to −50 HU for VAT, −190 to −30 HU for SAT and IMAT, and −29 to 150 HU for muscle) were maintained for body composition analysis. The option to save pictures in jpeg format of each image after segmentation was also selected. Segmental analysis for CSA and density estimates of each tissue using the test method took approximately 350 milliseconds per CT image. After the analysis, the segmentation of CSA for muscle, VAT, SAT, and IMAT was visually inspected using the picture files produced by the test method to evaluate if any of the images were incompletely segmented (defined as absence of color tagging or erroneous color tagging). The program neatly provides pictures (i.e., jpeg files) of all CT images in one folder, which enables the visual inspection of images at one time using Windows Explorer. Visual inspection took approximately 10 min to complete for the 420 images.

### 2.4. Statistical Analyses

Categorical variables were presented as percentage frequencies, with continuous variables presented as means ± standard deviations. Correlation between the two methods was evaluated using Lin’s concordance correlation coefficient (CCC) [12], intraclass correlation coefficient (ICC), and Spearman correlation coefficient. Agreement between the AutoMATiCA and human-based manual analysis technique were evaluated using: Dice similarity coefficient (DSC) and Bland–Altman plots [13]. The DSC values were compared across disease states, sex, and race/ethnicity using Mann–Whitney U test or Kruskal–Wallis Test. A score of ‘1’ for DSC implies perfect agreement, whereas a score of ‘0’ indicates no overlap or agreement. Bland–Altman plots were used to evaluate agreement between the two methods and to also determine limits of agreement (LOA) within 1.96 standard deviations. Statistical significance was defined as *p* < 0.05. Analyses were performed with SAS v9.4 (SAS Institute, Cary, NC, USA).

## 3. Results

### 3.1. Patient Characteristics

The sample consisted of 231 female patients (55%) and 187 male patients with various diagnoses including patients designated as healthy adults (*n* = 128) with acute illness (i.e., abdominal pain, hernia work-up) or receiving pre-surgical evaluations (i.e., organ donor, elective surgery work-up), patients with colorectal cancer (*n* = 127), and patients with metastatic breast cancer (*n* = 92), as shown in Table 1. Most patients were between 40–65 years of age (62%) and identified as either non-Hispanic Black (53%) or White (40%). The sample was mostly overweight (35%), followed by obese (34%) and low-normal (30%) BMI.

### 3.2. Body Composition Comparisons

The muscle, VAT, SAT, and IMAT CSA and tissue densities for the test and reference methods are shown in Table 2. Briefly, the muscle CSA for the test method versus the reference method was 143.19 ± 36 cm^2^ vs. 139.34 ± 37.91 cm^2^, respectively (Table 2). The VAT CSA for the test method was 122.08 ± 95.75 cm^2^ vs. 117.82 ± 93.56 cm^2^. The SAT CSA for the test method was 226.32 ± 142.55 cm^2^ vs. 215.41 ± 140.76 cm^2^ for the reference method. The muscle density for the test method was 35.52 ± 10.83 HU compared to 37.43 ± 16.34 HU for the reference method and for VAT HU it was −86.95 ± 9.83 HU vs. −87.10 ± 13.18 HU, respectively.

### 3.3. Correlation and Agreement Comparisons

Table 3 highlights the correlation and agreement between the test reference method using agreement statistics. The CCC, ICC, and Spearman’s correlation for muscle, VAT, SAT were all greater than 0.80, indicating strong positive correlations. The DSC estimates reflecting the overall accuracy of the test method compared to the reference for CSA and densities was lowest for IMAT CSA (0.83 ± 0.15) and highest for VAT density (0.99 ± 0.07) demonstrating good to almost perfect agreement.

### 3.4. Agreement Comparisons by Subgroups

**Figure 1:**
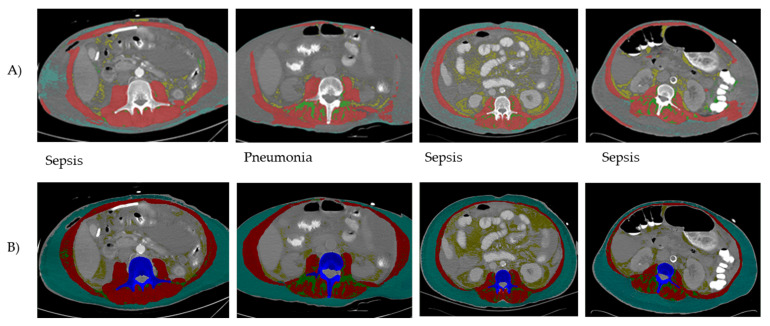
Examples of CT images following AutoMATiCA (test method) analysis (**A**) compared to semi-automated SliceOmatic (reference method) analysis (**B**). Discrepancies in tagging (i.e., coloring of different tissues) by the test method were noticed for all the abdominal tissues at this landmark (skeletal muscle and adipose tissues). The inconsistent performance of the test method was noted mainly in patients with a critical illness diagnosis.

The lowest DSC estimates were observed for patients with critical illness (*n* = 37) as shown in Table 4. The lowest DSC value observed in these patients was 0.65 for VAT CSA and highest for muscle CSA (0.87). In comparison, DSC estimates for all other diagnoses including metastatic breast cancer ranged between 0.91 (good agreement) to 0.99 (perfect agreement) excluding critical illness. Small (DSC ≤ 0.15) yet statistically significant differences in DSC scores were observed across diagnosis categories with critical illness having the largest difference for VAT CSA (DSC = 0.33). Per visual inspection of analyzed images by test method, a total of 12 of 28 images were incompletely segmented with the test method (Figure 1).

DSC estimates for muscle CSA and tissue densities stratified by sex and race/ethnicity remained in almost perfect agreement (DSC ≥ 0.97) with the exception of IMAT CSA (Table 5 and Supplemental Table S1 and Table S2). The IMAT CSA had the lowest DSC estimates for sex (females: 0.85 ± 0.15; males 0.80 ± 0.13), race/ethnicity (Blacks, *n* = 223: 0.82 ± 0.14; Whites, *n* = 169: 0.85 ± 0.15; Other, *n* = 28: 0.85 ± 0.18), and sex and race/ethnic categories as observed for the full sample. For BMI categories, the lowest DSC estimates were shown for IMAT CSA (DSC 0.82–0.84) compared to near perfect agreement for other body composition parameters (DSC > 0.90), as shown in Supplemental Table S3 (Supplemental Materials). The DSC for other parameters were consistent with strong to perfect agreement. Additionally, small (DSC ≤ 0.21) but statistically significant differences in DSC scores were observed by sex, race, sex and race/ethnic categories, and by BMI. As before, DSC estimates for remaining body composition by subgrouping consistent with good (>0.80) for IMAT CSA to near perfect agreement for other CSAs and densities (DSC 0.91–0.99).

**Figure 2:**
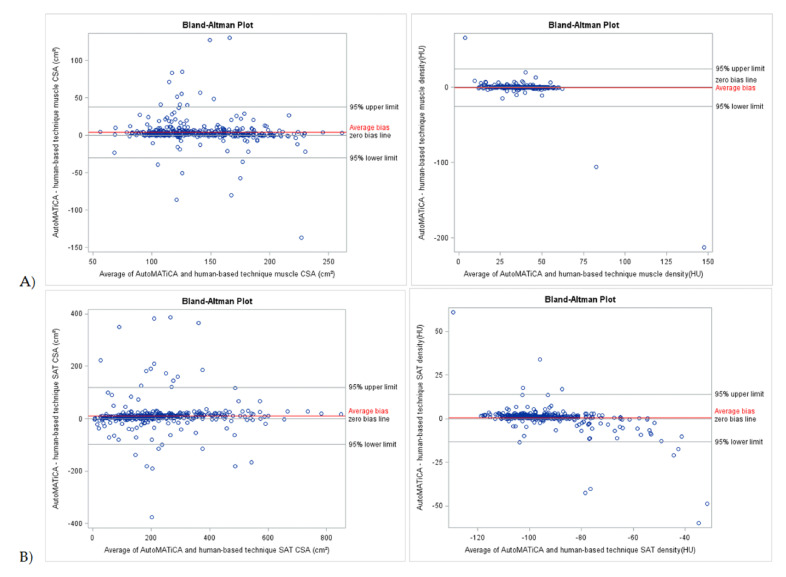
Bland-Altman plots of body composition parameters between test (AutoMATica) and reference (SliceOmatic plus ABACS + manual correction) method for entire sample. Plots show cross-sectional area (CSA) and density for muscle (**A**) and subcutaneous adipose tissue (SAT, **B**). Limits of agreement within 1.96 standard deviations are shown with average bias (red line) for each plot.

**Figure 3:**
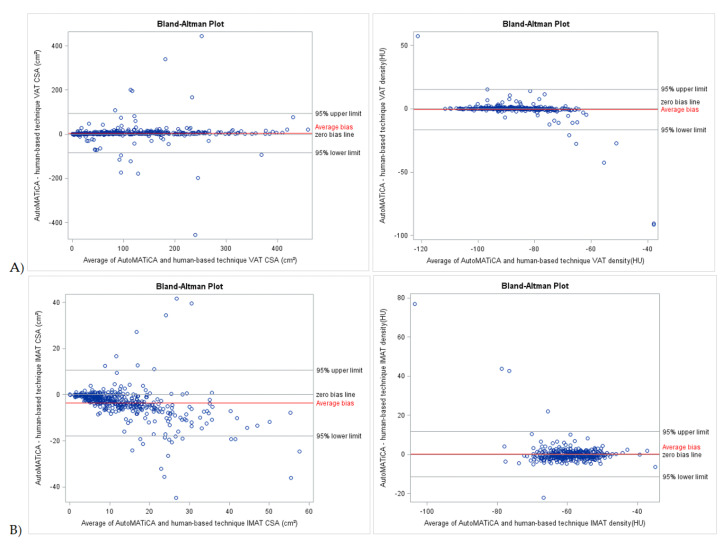
Bland-Altman plots of body composition parameters between test (AutoMATica) and reference (SliceOmatic plus ABACS + manual correction) method for entire sample. Plots show cross-sectional area (CSA) and density visceral adipose tissue (VAT, **A**), and intermuscular adipose tissue (IMAT, **B**). Limits of agreement within 1.96 standard deviations are shown with average bias (red line) for each plot.

Bland–Altman plots of CSA and densities for muscle, VAT, SAT, and IMAT showed narrow LOA of 3.85 [−29.99 and 37.70] cm^2^, 4.26 [−84.45 and 92.97] cm^2^, 10.91 [−97.98 and 119.79] cm^2^, −3.65 [7.30 and −17.95] cm^2^, −0.63 [−25.71 and 24.45] HU, −0.53 [−16.50 and 15.45] HU, 0.45 [−13.23 and 14.13] HU, 0.26 [−11.19 and 11.72] HU, respectively, supportive of good to excellent agreement with minimal proportional bias between the test and reference method for the full sample (Table 3, Figure 2 and Figure 3). Specific analysis of proportional bias revealed statistically significant bias for muscle CSA and muscle, VAT, SAT, and IMAT densities for the full sample (Table 3). The average bias line for IMAT CSA density was below the zero bias line, suggesting that, on average, the test method measurements are lower than the measurements estimated by the reference method.

Additional Bland–Altman and proportional bias analyses for sex, race, sex and race/ethnic categories and BMI are available in the Supplementary Materials. Proportional biases observed for the full sample remained for the same body composition parameters when explored by sex and race/ethnic categories with these exceptions: (1) proportional bias was not observed for muscle CSA and IMAT density in the Black subjects, and (2) proportional bias was not observed for muscle and IMAT CSA in the others category ( Supplemental Tables S4–S7).

## 4. Discussion

The validity of the test method (AutoMATiCA) was supported by the DSC scores for muscle, VAT, and SAT CSA at the L3 landmark, as observed in this study, which are consistent with estimates reported by others using automated segmentation neural networks [9,14,15]. These findings were consistent with Paris et al. (AutoMATiCA developer), who reported DCS estimates > 0.97 for muscle, VAT, and SAT and good accuracy for IMAT CSA (DSC > 0.88) [9]. Corresponding Bland–Altman plots of these body composition parameters revealed narrow LOA for the diverse cohort of patients evaluated in this study. Additionally, the lower DSC estimates for VAT, SAT, and IMAT CSA in this study were observed for the small cohort of critically ill patients (DSC > 0.65) but higher for muscle CSA (DSC > 0.87), suggesting better estimation of muscle CSA and overall good concordance between test and reference methods specific for this tissue. However, the DSC scores for muscle (0.87), IMAT (0.69), VAT (0.65), and SAT (0.69) for critically ill patients (*n*= 37) in the intensive care unit (ICU) in this study were much lower compared to scores reported for ICU patients (*n* = 30) by Paris et al., (IMAT DSC = 0.88; VAT DSC = 0.98; SAT DSC = 0.98) [9]. This difference in scores for IMAT, VAT, and SAT between the reference and test method suggests that CT images of critically ill patients analyzed using the test method (AutoMATiCA) should be reviewed post-analysis to determine which images may need to be reanalyzed. Critically ill patients often have fluid retention and anasarca, which can impact body composition analysis, which has been similarly suggested in patients with severe COVID-19 illness requiring hospitalization [16]. Thus, the presence of fluid retention and anasarca in critically ill patients may have limited the ability of the automated neural network, AutoMATiCA (test method), to differentiate tissue voxels in muscle and other tissues. Additionally, these data showed good to near perfect agreement (DSC > 0.80–0.99) between the two methods for most tissues including IMAT CSA following stratification by sex, BMI categories, race/ethnicity, and sex and race/ethnicity categories. Although small statistically significant differences in DSC scores were observed when the CT data were stratified by sex, race, and BMI, these estimates are likely clinically irrelevant. Such evidence further validates the utility of the test method for quick, efficient, accurate measurements of CSA and densities of abdominal tissues regardless of demographic characteristics.

Two recently published research studies also examine newer prototypes of automated artificial intelligence (AI) neural networks compared to human-based segmentation protocols for muscle, VAT, SAT, and IMAT CSA. Borrelli et al. examined the accuracy of an AI method to analyze CT images for muscle and SAT volume compared to a single CT image at the L3 landmark segmentation analysis (reference method) using a training group of 50 patients and a test group of 74 cancer patients providing two images each [14]. Their estimates of DSC for SAT (0.96, range 0.82–0.97) and muscle (0.94, range 0.82–0.97) volumes were similar to estimates for SAT CSA (0.93) and muscle CSA (0.97) for this study. Their findings support the use of their AI method for SAT and muscle volume estimation from CT images. Ackermans et al. also recently published a study testing the accuracy of a deep learning neural network trained on 3413 CT images for VAT, SAT, and muscle segmentation at the L3 vertebra compared against manual segmentation by a trained investigator [15]. The deep learning neural network was tested on 233 patients. Their results showed a median DSC of 0.93 (range 0.86–0.96) for muscle CSA, 0.95 (range 0.89–0.97) for VAT CSA, and 0.95 (range 0.92–0.96) for SAT CSA between the manual segmentation and newly developed deep learning neural network. These DSC scores reported by Ackermans et al. also aligned with the DSC scores obtained in this study for SAT and muscle CSA as stated above and for VAT CSA (DSC = 0.92). These data also support the neural network developed by Ackermans et al. for CSA body composition analysis of muscle, VAT, and SAT at the L3 landmark. Similar to the AutoMATiCA neural network tested in the present study, the AI-based neural networks by Borrelli et al. and Ackermans et al. provide reliable and quick analysis of body composition parameters at the L3 landmark [14,15] as well as volume estimates [15]. However, an additional benefit of using the test method (AutoMATiCA) developed by Paris et al. [9] that was validated in this study, aside from it being open-source, is that it also provides estimates for IMAT and VAT as well as estimates of tissue density unlike the neural networks developed by Borrelli et al. and Ackermans et al.

## 5. Conclusions

The test method shows good performance and almost perfect concordance for body composition analysis at L3 per DSC estimates and Bland–Altman plots for muscle, VAT, SAT, and IMAT for most clinical populations evaluated in this study. Care must be taken to assess the density of the CT images from critically ill patients before applying the automated neural network (test method). An important next step would be determining what is it about the CT images from critically ill patients at the L3 region that makes them less compatible with this neural network. Once this is determined, future studies using larger, more diverse clinical populations, particularly for critically ill patients, are needed to confirm the utility, accuracy, and generalizability of the automated neural network (AutoMATiCA) tested herein.

## Figures and Tables

**Table 1:** Patient Characteristics.

Variable	Levels	N (%)
**Age Group**		
	Young: <40 years	20 (4.76)
	Middle: 40–65 years	263 (62.62)
	Older: >65 years	135 (32.14)
	Unknown	2 (0.48)
**BMI Group**		
	Low/Normal: <25.0 kg/m^2^	127 (30.24)
	Overweight: 25–29.9 kg/m^2^	149 (35.48)
	Obese: >30.0 kg/m^2^	142 (33.81)
	Missing	2 (0.48)
**Sex**		
	Female	231 (55.00)
	Male	187 (44.52)
	Unknown	2 (0.48)
**Race/Ethnicity**		
	Black	223 (53.10)
	White	169 (40.24)
	Other	28 (6.67)
**Diagnosis**		
	Healthy Adults	128 (30.48)
	Colorectal Cancer	127 (30.24)
	Metastatic Breast Cancer	92 (21.90)
	Critical Illness	37 (8.81)
	COVID-19	22 (5.24)
	Early-stage Breast Cancer	14 (3.33)

BMI: body mass index.

**Table 2:** Summary of body composition parameters following segmental tissue analysis using test and reference methods.

Body Composition Parameter	Automated Program—AutoMATiCA (Test Method)								Human-Based Sliceomatic (Reference Method)							
	N	Mean	Std Dev	Median	Lower Quartile	Upper Quartile	Min.	Max.	N	Mean	Std Dev	Median	Lower Quartile	Upper Quartile	Min.	Max.
Muscle area	420	143.19	36.00	137.39	114.50	169.24	56.57	262.79	420	139.34	37.91	131.60	109.60	165.05	54.02	295.10
VAT area	420	122.08	95.75	96.28	46.90	171.65	0.56	474.85	420	117.82	93.56	89.98	46.38	167.40	0.03	468.20
SAT area	420	226.32	142.55	192.24	124.80	299.91	2.56	857.65	420	215.41	140.76	184.10	118.40	282.70	−84.15	841.30
IMAT area	420	12.34	9.12	9.85	6.09	15.64	0.10	51.45	398	15.61	11.58	12.11	7.26	20.19	0.01	73.44
Muscle density	420	35.52	10.83	36.21	28.36	43.77	3.63	61.10	383	37.43	16.34	37.29	29.82	44.73	−29	254.10
VAT density	420	−86.95	9.83	−88.02	−93.51	−79.76	−111.56	−61	383	−87.1	13.18	−88.92	−94.26	−81.28	−150	7.59
SAT density	420	−93	14.43	−96.64	−102.62	−87.05	−118.28	−44.23	383	−94.76	16.21	−98.42	−104.2	−90.03	−160	−4.93
IMAT density	420	−58.75	6.32	−58.45	−62.84	−54.5	−79.38	−36.16	361	−59.23	8.03	−58.8	−62.88	−54.81	−142	−31.67

Muscle Area: Cross-sectional area (CSA) for muscle using AutoMATiCA (variable: Muscle_CSA) or Sliceomati (variable: SM) program; VAT area: CSA for visceral adipose tissue (VAT) using AutoMATiCA (variable: VAT_CSA) or Sliceomatic (variable: VAT) program; SAT area: CSA for subcutaneous adipose tissue (SAT) using AutoMATiCA (variable: SAT_CSA) or Sliceomatic (variable: SAT) program; IMAT area: CSA for intermuscular adipose tissue (IMAT)using AutoMATiCA (variable: IMAT_CSA) or Sliceomatic (variable: IMAT) program; Muscle density: Density (proxy for ‘quality of tissue’) defined by mean CT Hounsfield unit (HU) of L3 muscle groups using Automatica (variable: Muscle_HU) or Sliceomatic (variable: SMHU) program; VAT density: VAT density defined by mean CT Hounsfield unit (HU) using Automatica software (variable: VAT_HU) or Sliceomatic (variable: VATHU) program; SAT density: SAT density defined by mean CT Hounsfield unit (HU) using Automatica software (variable: SAT_HU) or Sliceomatic (variable: SATHU) program; IMAT density: IMAT density defined by mean CT Hounsfield unit (HU) using Automatica software (variable: IMAT_HU) or Sliceomatic (variable: IMATHU) program.

**Table 3:** Summary of correlation and agreement statistics for patient cohort measured by Lin’s concordance correlation coefficient, intra-class correlation, Spearman correlation coefficients, and Bland-Altman summary statistics including assessment of proportional bias using Pearson correlation coefficients.

Comparisons		N	Lin’s Concordance Correlation Coefficient	Intraclass Correlation	Spearman Correlation Coefficients	Dice Similarity Coefficient (DSC)		Bland-Altman						
Automatic Program–AutoMATiCA	Human-Based Sliceomatic							Bland-Altman Plots (Difference between AutoMATiCA and Human-Based Technique)				Proportional Bias		
(Test Method– Autosegmentation)	(Reference Method)					Mean	SD	Mean	SD	Lower LOA	Upper LOA	Pearson Correlation Coefficients	*p*-Value	Performance
Muscle CSA	SM	420	0.89	0.89	0.9	0.97	0.06	3.85	17.27	−29.99	37.7	−0.11	0.02	Proportional bias
VAT CSA	VAT	420	0.89	0.89	0.9	0.92	0.17	4.26	45.26	−84.45	92.97	0.05	0.31	No Proportional bias
SAT CSA	SAT	420	0.92	0.92	0.91	0.93	0.14	10.91	55.56	−97.98	119.79	0.03	0.5	No Proportional bias
IMAT CSA	IMAT	398	0.75	0.76	0.83	0.83	0.15	−3.65	7.3	−17.95	10.65	−0.42	<0.00	Proportional bias
Muscle HU	SMHU	383	0.54	0.56	0.96	0.98	0.06	−0.63	12.8	−25.71	24.45	−0.54	<0.00	Proportional bias
VAT HU	VATHU	383	0.73	0.75	0.97	0.99	0.07	−0.53	8.15	−16.5	15.45	−0.48	<0.00	Proportional bias
SAT HU	SATHU	383	0.8	0.9	0.95	0.98	0.07	0.45	6.98	−13.23	14.13	−0.49	<0.00	Proportional bias
IMAT HU	IMATHU	361	0.64	0.67	0.9	0.98	0.03	0.26	5.84	−11.19	11.72	−0.37	<0.00	Proportional bias

Muscle CSA: Cross-sectional area for muscle; VAT CSA: Cross-sectional area for visceral adipose tissue (VAT); SAT CSA: Cross-sectional area for subcutaneous adipose tissue (SAT); IMAT CSA: Cross-sectional area for intermuscular adipose tissue (IMAT); Muscle HU: Mean HU (or density) for muscle; VAT HU: Mean HU (or density) for visceral adipose tissue (VAT); SAT HU: Mean HU (or density) for subcutaneous adipose tissue (SAT); IMAT HU: Mean HU (or density) for intermuscular adipose tissue (IMAT); LOA: Limits of agreement.

**Table 4:** Dice similarity coefficient estimates across diagnosis.

Body Composition Parameters	ALL (*n* = 420)	Healthy Adults (*n* = 128)			Colorectal Cancer (*n* = 127)			Metastatic Breast Cancer (*n* = 92)			Critical Illness (*n* = 37)			COVID-19 (*n* = 19)			Breast Cancer (*n* = 14)			*p*-Value *
		N	Mean	SD	N	Mean	SD	N	Mean	SD	N	Mean	SD	N	Mean	SD	N	Mean	SD	
**Muscle CSA**	0.97	128	0.99	0.04	127	0.99	0.02	92	0.97	0.03	37	0.87	0.12	22	0.96	0.06	14	0.99	0.01	<0.00
**VAT CSA**	0.92	128	0.95	0.12	127	0.95	0.11	92	0.92	0.15	37	0.65	0.33	22	0.99	0.02	14	0.96	0.05	<0.00
**SAT CSA**	0.93	128	0.95	0.1	127	0.95	0.07	92	0.96	0.07	37	0.69	0.28	22	0.99	0.01	14	0.98	0.01	<0.00
**IMAT CSA**	0.83	128	0.8	0.1	127	0.84	0.1	92	0.91	0.13	37	0.67	0.26	0			14	0.91	0.08	<0.00
**Muscle HU**	0.98	128	0.99	0.03	127	0.99	0.02	92	0.96	0.11	0			22	0.94	0.1	14	0.99	0	0.01
**VAT HU**	0.99	128	0.99	0.05	127	0.99	0.02	92	0.97	0.12	0			22	1	0.01	14	1	0	<0.00
**SAT HU**	0.98	128	0.97	0.11	127	0.99	0.03	92	0.98	0.04	0			22	1	0	14	1	0	<0.00
**IMAT HU**	0.98	128	0.98	0.03	127	0.99	0.02	92	0.98	0.04	0			0			14	0.99	0.01	0.43

* Kruskal-Wallis test statistic; Muscle CSA: Cross-sectional area for muscle; VAT CSA: Cross-sectional area for visceral adipose tissue (VAT); SAT CSA: Cross-sectional area for subcutaneous adipose tissue (SAT); IMAT CSA: Cross-sectional area for intermuscular adipose tissue (IMAT); Muscle HU: Mean HU (or density) for muscle; VAT HU: Mean HU (or density) for visceral adipose tissue (VAT); SAT HU: Mean HU (or density) for subcutaneous adipose tissue (SAT); IMAT HU: Mean HU (or density) for intermuscular adipose tissue (IMAT).

**Table 5:** DSC by Sex and Race/ethnic categories.

	ALL (*n* = 418)	Female–Black (*n* = 112)			Female–Other (*n* = 16)			Female–White (*n* = 103)			Male–Black (*n* = 111)			Male–Other (*n* = 10)			Male–White (*n* = 66)			
		N	Mean	SD	N	Mean	SD	N	Mean	SD	N	Mean	SD	N	Mean	SD	N	Mean	SD	*p*-Value *
Muscle CSA	0.97	112	0.97	0.05	16.00	0.95	0.10	103.00	0.97	0.05	111.00	0.98	0.04	10.00	0.93	0.11	66.00	0.98	0.06	<0.00
VAT CSA	0.92	112	0.91	0.19	16.00	0.93	0.20	103.00	0.91	0.18	111.00	0.92	0.16	10.00	0.87	0.23	66.00	0.95	0.13	0.04
SAT CSA	0.93	112	0.93	0.15	16.00	0.90	0.21	103.00	0.95	0.10	111.00	0.93	0.12	10.00	0.86	0.29	66.00	0.94	0.13	0.00
IMAT CSA	0.83	103	0.83	0.15	14.00	0.85	0.20	103.00	0.87	0.15	109.00	0.81	0.13	4.00	0.80	0.15	63.00	0.81	0.14	<0.00
Muscle HU	0.98	102	0.98	0.05	15.00	0.91	0.19	96.00	0.98	0.07	104.00	0.99	0.02	6.00	0.99	0.02	58.00	0.98	0.03	0.88
VAT HU	0.99	102	0.99	0.03	15.00	0.94	0.21	96.00	0.98	0.09	104.00	0.99	0.04	6.00	0.99	0.01	58.00	0.99	0.03	0.01
SAT HU	0.98	102	0.98	0.09	15.00	0.98	0.07	96.00	0.99	0.03	104.00	0.97	0.08	6.00	1.00	0.01	58.00	0.98	0.04	<0.00
IMAT HU	0.98	93	0.99	0.01	13.00	0.97	0.07	96.00	0.98	0.03	102.00	0.98	0.02	0.00			55.00	0.98	0.05	0.77

* Kruskal-Wallis Test; Muscle CSA: Cross-sectional area for muscle; VAT CSA: Cross-sectional area for visceral adipose tissue (VAT); SAT CSA: Cross-sectional area for subcutaneous adipose tissue (SAT); IMAT CSA: Cross-sectional area for intermuscular adipose tissue (IMAT); Muscle HU: Mean HU (or density) for muscle; VAT HU: Mean HU (or density) for visceral adipose tissue (VAT); SAT HU: Mean HU (or density) for subcutaneous adipose tissue (SAT); IMAT HU: Mean HU (or density) for intermuscular adipose tissue (IMAT).

## Data Availability

No new data were created or analyzed in this study. Data sharing is not applicable to this article.

## Supplementary Materials

The following supporting information can be downloaded at: https://www.mdpi.com/article/10.3390/s22093357/s1, Table S1: DSC by Sex; Table S2: DSC by Race/Ethnic categories; Table S3: DSC by BMI; Table S4: Bland–Altman and proportional bias statistics for females; Table S5: Bland–Altman and proportional bias statistics for males; Table S6: Bland–Altman and proportional bias statistics for Black race/ethnic category; Table S7: Bland–Altman and proportional bias statistics for White race/ethnic category; Table S8: Bland–Altman and proportional bias statistics for Others race/ethnic category.
