# Supplementary material for: Concordance of Computed Tomography Regional Body Composition Analysis Using a Fully Automated Open-Source Neural Network versus a Reference Semi-Automated Program with Manual Correction
Source: Sensors (Basel). 2022 Apr 27;22(9):3357. doi: 10.3390/s22093357 (PMC9101564; doi:10.3390/s22093357)
Supplement: Supplementary file 1 [file sensors-22-03357-s001.zip › sensors-1643241-supplementary.pdf]

Online Supplementary Materials

Title: Concordance of computed tomography regional body composition analysis using fully automated open-source framework versus a reference semi-automated program with manual correction

Authors:[1]Gomez-Perez, Sandra, PhD, RDN, [2]Zhang, Yanyu, MS, [3]Byrne, Cecily, MS, RDN, [4]Wakefield, Connor, MD, [5]Geesey, Thomas, MD, [5]Sclamberg, Joy, MD, [1]Peterson, Sarah, PhD, RDN

Affiliations:

[1] Rush University, Department of Clinical Nutrition, Chicago, IL, USA

[2] Rush University Medical Center, Rush Bioinformatics and Biostatistics Core, Chicago, IL, USA

[3] University of Illinois at Chicago, Department of Kinesiology and Nutrition, Chicago, IL, USA

[4] Brooke Army Medical Center, Department of Internal Medicine, Fort Sam Houston, TX, USA

[5] Rush University Medical Center, Department of Radiology, Chicago, IL, USA

Corresponding Author: Sandra Gomez-Perez, PhD, RDN; Rush University Medical Center, Department of Clinical Nutrition, Armour Academic Center, 600 S. Paulina St, AAC 737D, Chicago, IL, 60612; email: sandra_l_gomez-perez@rush.edu

Key Words: agreement, computed tomography, automated segmentation, regional body composition, abdominal tissues

Table S1. DSC by Sex

	ALL (n=418)	Female (n=231)			Male (n=187)			P-value*
		N	Mean	SD	N	Mean	SD	
Muscle CSA	0.97	231	0.97	0.06	187.00	0.98	0.06	0.00
VAT CSA	0.92	231	0.91	0.19	187.00	0.92	0.15	0.12
SAT CSA	0.93	231	0.94	0.14	187.00	0.93	0.14	0.00
IMAT CSA	0.83	220	0.85	0.15	176.00	0.81	0.13	<.0001
Muscle HU	0.98	213	0.98	0.08	168.00	0.99	0.02	0.69
VAT HU	0.99	213	0.98	0.08	168.00	0.99	0.04	0.01
SAT HU	0.98	213	0.98	0.07	168.00	0.98	0.07	<.0001
IMAT HU	0.98	202	0.98	0.03	157.00	0.98	0.03	0.27

*Mann-Whitney U Test

Table S2. DSC by Race/Ethnic categories

	ALL (n=420)	Black (n=223)			Other (n=28)			White (n=169)			P-value*
		N	Mean	SD	N	Mean	SD	N	Mean	SD	
Muscle CSA	0.97	223	0.98	0.05	28.00	0.94	0.10	169.00	0.97	0.06	<.0001
VAT CSA	0.92	223	0.91	0.18	28.00	0.91	0.20	169.00	0.93	0.16	0.36
SAT CSA	0.93	223	0.93	0.13	28.00	0.89	0.23	169.00	0.94	0.11	0.21
IMAT CSA	0.83	212	0.82	0.14	20.00	0.85	0.18	166.00	0.85	0.15	0.00
Muscle HU	0.98	206	0.99	0.04	23.00	0.93	0.16	154.00	0.98	0.06	0.35
VAT HU	0.99	206	0.99	0.04	23.00	0.96	0.17	154.00	0.99	0.07	0.45
SAT HU	0.98	206	0.98	0.08	23.00	0.98	0.05	154.00	0.98	0.04	0.00
IMAT HU	0.98	195	0.99	0.02	15.00	0.97	0.07	151.00	0.98	0.04	0.95

*Kruskal-Wallis Test

Table S3. DSC by BMI

	ALL (n=418)	Low/Normal (n=127)			Overweight (n=149)			Obese (n=142)			P-value*
		N	Mean	SD	N	Mean	SD	N	Mean	SD	
Muscle CSA	0.97	127	0.97	0.04	149.00	0.98	0.06	142.00	0.97	0.07	0.00
VAT CSA	0.92	127	0.90	0.17	149.00	0.93	0.17	142.00	0.92	0.18	0.00
SAT CSA	0.93	127	0.91	0.18	149.00	0.95	0.09	142.00	0.94	0.13	0.03
IMAT CSA	0.83	121	0.84	0.15	143.00	0.83	0.13	132.00	0.82	0.16	0.29
Muscle HU	0.98	116	0.99	0.06	141.00	0.98	0.07	124.00	0.98	0.05	0.43
VAT HU	0.99	116	0.98	0.09	141.00	0.99	0.07	124.00	0.99	0.02	0.00
SAT HU	0.98	116	0.97	0.11	141.00	0.98	0.03	124.00	0.99	0.02	0.00
IMAT HU	0.98	110	0.98	0.03	135.00	0.98	0.03	114.00	0.98	0.04	0.76

*Kruskal-Wallis Test

Table S4. Bland – Altman and proportional bias statistics for females

Comparisons			Bland-Altman						
			Bland-Altman plot (difference between AutoMATiCA and human-based technique)				Proportional bias		
Test Method	Reference Method	N	mean	SD	lower	upper	Pearson correlation coefficient	P-value	
Muscle CSA	SM	231	5.68	14.95	-23.63	35.00	0.09	0.17	No Proportional bias
VAT CSA	VAT	231	5.62	55.70	-103.55	114.80	0.11	0.10	No Proportional bias
SAT CSA	SAT	231	15.97	63.01	-107.53	139.46	0.02	0.71	No Proportional bias
IMAT CSA	IMAT	220	-2.92	8.32	-19.23	13.40	-0.34	<.0001	Proportional bias
Muscle HU	SMHU	213	-1.30	16.49	-33.61	31.01	-0.67	<.0001	Proportional bias
VAT HU	VATHU	213	-0.69	9.44	-19.19	17.80	-0.56	<.0001	Proportional bias
SAT HU	SATHU	213	0.22	6.52	-12.56	13.00	-0.43	<.0001	Proportional bias
IMAT HU	IMATHU	202	0.37	4.99	-9.41	10.15	-0.25	0.00	Proportional bias

Table S5. Bland – Altman and proportional bias statistics for males

Comparisons			Bland-Altman						
			Bland-Altman plot (difference between AutoMATiCA and human-based technique)				Proportional bias		
Test Method	Reference Method	N	Mean	SD	lower	upper	Pearson correlation coefficient	P-value	
Muscle CSA	SM	187	1.58	19.64	-36.92	40.08	-0.14	0.05	No Proportional bias
VAT CSA	VAT	187	2.63	27.80	-51.86	57.13	0.03	0.71	No Proportional bias
SAT CSA	SAT	187	4.61	44.39	-82.39	91.60	-0.04	0.56	No Proportional bias
IMAT CSA	IMAT	176	-4.61	5.69	-15.76	6.54	-0.57	<.0001	Proportional bias
Muscle HU	SMHU	168	0.20	5.31	-10.22	10.61	-0.26	0.00	Proportional bias
VAT HU	VATHU	168	-0.31	6.23	-12.53	11.90	-0.42	<.0001	Proportional bias
SAT HU	SATHU	168	0.76	7.56	-14.07	15.58	-0.56	<.0001	Proportional bias
IMAT HU	IMATHU	157	0.15	6.83	-13.23	13.54	-0.45	<.0001	Proportional bias

Table S6. Bland–Altman and proportional bias statistics for Black race/ethnic category

Comparisons			Bland-Altman						
			Bland-Altman plot (difference between AutoMATiCA and human-based technique)				Proportional bias		
Test Method	Reference Method	N	Mean	SD	lower	upper	Pearson correlation coefficient	P-value	
Muscle CSA	SM	223	2.92	13.56	-23.67	29.50	-0.06	0.38	No Proportional bias
VAT CSA	VAT	223	4.23	51.11	-95.95	104.40	0.00	0.96	No Proportional bias
SAT CSA	SAT	223	11.35	52.14	-90.84	113.54	0.05	0.47	No Proportional bias
IMAT CSA	IMAT	212	-3.96	7.01	-17.71	9.79	-0.36	<.0001	Proportional bias
Muscle HU	SMHU	206	-0.13	1.66	-3.39	3.13	-0.32	<.0001	Proportional bias
VAT HU	VATHU	206	-0.68	4.74	-9.97	8.61	-0.47	<.0001	Proportional bias
SAT HU	SATHU	206	0.04	6.62	-12.94	13.03	-0.57	<.0001	Proportional bias
IMAT HU	IMATHU	195	-0.33	2.66	-5.55	4.88	0.00	0.98	No Proportional bias

Table S7. Bland–Altman and proportional bias statistics for White race/ethnic category

Comparisons			Bland-Altman						
			Bland-Altman plot (difference between AutoMATiCA and human-based technique)				Proportional bias		
Test Method	Reference Method	N	Mean	SD	lower	upper	Pearson correlation coefficient	P-value	
Muscle CSA	SM	169	4.60	18.25	-31.16	40.36	-0.18	0.02	Proportional bias
VAT CSA	VAT	169	4.16	36.49	-67.37	75.69	0.15	0.05	No Proportional bias
SAT CSA	SAT	169	7.89	48.12	-86.43	102.21	0.08	0.29	No Proportional bias
IMAT CSA	IMAT	166	-3.38	7.56	-18.20	11.44	-0.49	<.0001	Proportional bias
Muscle HU	SMHU	154	-0.04	10.40	-20.42	20.34	-0.41	<.0001	Proportional bias
VAT HU	VATHU	154	0.16	9.01	-17.51	17.83	-0.46	<.0001	Proportional bias
SAT HU	SATHU	154	1.26	7.13	-12.72	15.25	-0.40	<.0001	Proportional bias
IMAT HU	IMATHU	151	0.75	7.73	-14.39	15.89	-0.52	<.0001	Proportional bias

Table S8. Bland–Altman and proportional bias statistics for Others race/ethnic category

Comparisons			Bland-Altman						
			Bland-Altman plot (difference between AutoMATiCA and human-based technique)				Proportional bias		
Test Method	Reference Method	N	mean	SD	lower	upper	Pearson correlation coefficient	P-value	
Muscle CSA	SM	169	4.60	18.25	-31.16	40.36	-0.18	0.02	Proportional bias
VAT CSA	VAT	169	4.16	36.49	-67.37	75.69	0.15	0.05	No Proportional bias
SAT CSA	SAT	169	7.89	48.12	-86.43	102.21	0.08	0.29	No Proportional bias
IMAT CSA	IMAT	166	-3.38	7.56	-18.20	11.44	-0.49	<.0001	Proportional bias
Muscle HU	SMHU	154	-0.04	10.40	-20.42	20.34	-0.41	<.0001	Proportional bias
VAT HU	VATHU	154	0.16	9.01	-17.51	17.83	-0.46	<.0001	Proportional bias
SAT HU	SATHU	154	1.26	7.13	-12.72	15.25	-0.40	<.0001	Proportional bias
IMAT HU	IMATHU	151	0.75	7.73	-14.39	15.89	-0.52	<.0001	Proportional bias
